# Prevalence and associated factors depressive symptoms among healthcare professionals at Dessie comprehensive specialized hospital, Ethiopia

**DOI:** 10.1186/s12888-022-04102-y

**Published:** 2022-07-04

**Authors:** Asmare Belete, Tamrat Anbesaw

**Affiliations:** Department of Psychiatry, College of Medicine and Health Science, Wollo University, P.O. Box 1145, Dessie, Ethiopia

**Keywords:** Depressive symptom, Healthcare professionals, Dessie, Ethiopia

## Abstract

**Background:**

Depression among healthcare professionals results in adverse effects which might include decreased impairment of work performance, an increased turnover rate, and vulnerability to clinical error. Despite that, there is a paucity of information concerning depression among healthcare professionals in Ethiopia. This study aimed to assess the prevalence and identifying the associated factors of depression among health care professionals working at Dessie Comprehensive specialized hospital, Ethiopia.

**Method:**

A cross-sectional study was conducted on 252 healthcare professionals at Dessie Comprehensive Specialized Hospital, Northeast, Ethiopia. They were randomly selected and depression was measured by the Patient Health Questionnaire (PHQ-9) with a score of 5 and above. A multivariable logistic regression analysis was used to explore the potential determinants of depressive symptoms among the participants. A *p*-value less than 0.05 was considered significant and, adjusted OR (AOR) with 95% CI was used to present the strength of the association.

**Result:**

The prevalence of depressive symptoms among healthcare professionals was 27.8% (95% CI: 22.6,33.7). Among participants who had reported depressive symptoms, 72.2, 20.2, 6, and 1.6% reported no, mild, moderate, and severe depressive symptoms, respectively. In multivariable analysis, being female (AOR = 1.94; 95% CI: 1.12,3.67), unmarried (AOR = 2.16; 95% CI: 1.12,4.15), having a family history of mental illness (AOR = 7.31; 95% CI: 2.27,23.49), and current substance use (AOR = 2.67; 95% CI: 1.36,5.24) were found to be significant predictors of depressive symptoms.

**Conclusion:**

Depressive symptoms were highly prevalent among primary health care professionals. Being female, unmarried, family history of mental illness, and current substance use had a significant association with depressive symptoms among healthcare professionals. They should be promptly screened and managed at a healthcare institution.

## Background

Depression is a common mental disorder that presents with depressed mood, loss of pleasure or interest, decrease in energy, feeling of guilt or low self-worth, disturbed sleep and appetite, and poor concentration [1]. According to the WHO, depression is a common mental disorder that is a possible leading cause of disability [2]. The prevalence of depressive symptoms varies from region to region across the world population, varies between high-income, middle-income, and low-income countries, and ranges approximately in most countries between 3 to 16.9% [3]. It is estimated that depressive symptoms would become the second leading cause of Disability Adjusted Life Years (DALYs) by the year 2030 [4]. In Ethiopia depression contribute to about 6.5% of the burden of disease [5].

Depression often occurs as a result of adverse life events, such as the loss of a loved one or loss of health. However, it also causes problems without any apparent reason. This problem can become chronic or recurrent and lead to significant impairment in an individual’s ability to take care of their everyday activities [6]. Although healthy and highly functional employees are important to ensure the efficient delivery of any service, healthcare workers are thought to be at risk of developing depression due to the nature of their job [7]. Furthermore, depression results from a complex interaction of social, psychological, and biological factors in health care professionals [8]. Various studies on psychological morbidity amongst healthcare professionals have shown that they often experience stress, psychological distress, and depression at work [9]. This results in severely depressed individuals experiencing suicidal thoughts, plans, or even attempting suicide. The proportion of those attempting suicide was 2.3 times for females and 1.4 times for males when compared to the general population [9, 10].

Globally, various studies showed that the prevalence of depressive symptoms among healthcare professionals were in Australia 32.4% [11], China 38% [12], Hong Kong 35.8% [13], Vietnam 13.2% [14], Saudi Arabia 43.9% [15], Baghdad 70.25% [16], Egypt 59% [17], Malaysia 10.7% [18], Pakistan (24.8%) [19], and Nigeria 17.3% [20].

In addition, healthcare professionals are always demanding physically and mentally, which requires a careful and clever decision on life and death issues in a short period of time, with limited resources at hand, especially at the time of a medical emergency. Poor mental health among healthcare professionals hinders professional performance associated with medical errors, increases turnover, decreases clinical competency, and lowers the quality of the care provided by them, which will eventually negatively affect the quality of patient care and safety [21, 22].

There are several risk factors for depressive symptoms among healthcare professionals. Some of the factors associated with depressive symptoms are the clinical specialty, chronic illness, substance use, psychiatric disorder, insufficient social support, family history of mental illness, being unmarried, low income, excess working hours, taking responsibility for patients, being female, substance use, and working night shifts were found to be highly associated with the occurrence of mental distress like depressive symptoms among healthcare professionals [13, 17, 20, 23, 24].

This issue is rarely raised worldwide and also in Ethiopia, there is a limited study on the prevalence of depressive symptoms among healthcare professionals. To the best of the investigator’s knowledge, there are no published studies in Ethiopia on this subject. Therefore, this study aimed to assess the prevalence of depressive symptoms and identify the associated factors among healthcare professionals to fill the existing gap in the literature and used as baseline evidence for the program planner and strategic developer for the prevention and intervention of depression.

## Methods and materials

### Study area

The study was conducted at Dessie Comprehensive Specialized Hospital, Dessie, South Wollo zone, North-east, Ethiopia. It is located 401 km northeast of Addis Ababa, the capital city of Ethiopia, and 480 km away from Bahir Dar, which is the capital city of the Amhara region. The hospital provides many services, including preventive, curative, and rehabilitative care for patients coming from all woredas and zones of Eastern Amhara and Afar regional states. The hospital has a range of specialties, including pediatric, medical, surgical, ophthalmology, gynecology, and orthopedic. In total, the hospital has 563 staff, 240 beds, and more than 20 specialists in different departments to provide essential care.

### Study design and period

An Institutional based cross-sectional study was conducted from March 01–20/2020.

### Source population

All healthcare professionals who worked in Dessie Comprehensive Specialized Hospital.

### Study population

Healthcare professionals who worked in Dessie Comprehensive Specialized Hospital during the study period.

### Inclusion and exclusion criteria

#### Inclusion criteria

All healthcare professionals who worked at Dessie Comprehensive Specialized Hospital.

#### Exclusion criteria

Healthcare professionals who were on annual leave and seriously ill during data collection were excluded.

### Sample size determination and sampling technique

#### Sample size determination

The sample size was determined by using the single population proportion formula by considering the following assumptions, by assuming a 5% degree of freedom and 95% confidence interval at alpha (α =0.05), population proportion (50%), and correction formula because the total population was < 10,000 and 10% non-response rate and the result gave you sample size of 252.

#### Sampling technique

**Fig. 1:**
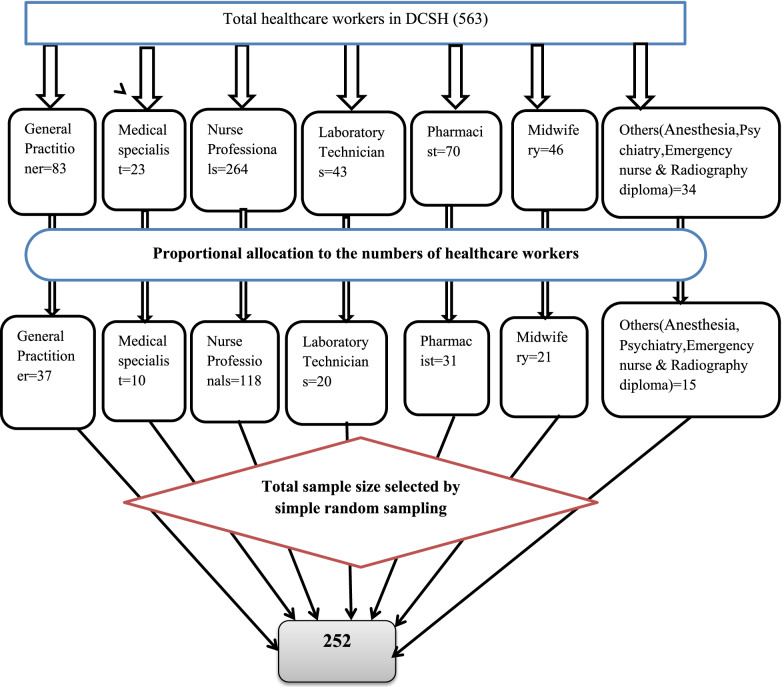
Proportional allocation to study the prevalence of depressive symptoms and associated factors among healthcare professionals working at Dessie Comprehensive Specialized Hospital Dessie, Ethiopia, 2020

The sampling technique employed stratified random sampling based on the type of profession (Fig. 1).

### Data collection method and tool

A self-administered questionnaire was used which has different subunits, questionnaires to assess socio-demographic factors, clinical related factors, psychosocial factors, and substance-related factors. The Patient Health Questionnaire-9 was used for the assessment of depressive symptoms, with a total score of the nine items ranging from 0 to 27. No depressive symptoms, mild, moderate, and severe depressive symptoms were defined by the tool as total scores of 0–4, 5–9, 10–14, and 15 and above, respectively [25]. Previously, the screening tool was validated in the Ethiopian population with a sensitivity of 86% and specificity of 67%, and those scoring 5 and above were defined as having depressive symptoms [26]. The internal consistency (Cronbach alpha) of (PHQ-9) in this study was 0.91. Based on the Oslo 3- item social support scale, those with a score of 3–8 were classified as poor social support, 9–11 as moderate social support, and 12–14 as strong social support [27]. The WHO student drug-use questionnaire was used to measure substance use [28]. The presence of family mental illness, chronic diseases such as hypertension, or others were assessed by self-report (dichotomous response of yes and no response).

### Data collection procedure

The questionnaire was prepared first in English and translated into the Amharic language then back-translated to English to check the consistency. Two days of training were given to the three data collectors (BSc in psychiatry) and one supervisor (Msc in psychiatry). The pre-test was conducted with 5% (*n* = 13) of the participants in the Dessie health center to identify potential problems in data collection tools and modification of the questionnaire. Regular supervision and support were given to data collectors by the supervisor and principal investigator. Data were checked for completeness and consistency by supervisors and principal investigators daily during data collection time for its completeness.

### Data processing and analysis

After the data were checked and cleaned, it was entered into the computer using Epi-data version 3.1 and it was exported to SPSS version 26 statistical software for further analysis. Independent variables with a *p*-value of < 0.25 in the bivariable model were included in the multivariable regression model to control for possible confounding effects. The model of fitness was checked by Hosmer and Lemeshow goodness. All variables with a *p*-value of < 0.05 in the multivariable model were considered statistically significant and the strength of the association was presented by an odds ratio of 95% C.I. Then the result was presented in the form of text, a table, or graph.

## Results

### Socio-demographic characteristics of the respondents

A total of 252 participants were involved in this study and the response rate was 100%. Out of the total participants, the majority of 163 (64.7%) were male. The mean age of the respondents was 34.85 (SD = ± 8.41 years), with a minimum and maximum age of 22 and 60 years, respectively. The majority of the participants, 148 (58.7%), 150 (59.5%), and 200 (79.4) were unmarried, Orthodox Christians, and Amhara, respectively. Most of the participants, 207 (82.1%), were degree holders, and 118 (46.8%) were nurses in their profession. We found, 108(42.9%) respondents’ service duration was between 6 and 11 years. Nearly two-thirds of 169 (67.1%) of the participants’ working hours were in the range of 38–44 hours per week. According to the participant’s responses, nearly one-third 83 (32.9%) respondents earned monthly income between the ranges of 8017–10,170 ETB (Table 1).

**Table 1:** Socio-demographic characteristics of healthcare professionals at DCSH, Dessie, Northeast, Ethiopia, 2020 (*N* = 252)

Variables	Category	Frequency	Percentage (%)
Sex	Male	163	64.7
	Female	89	35.3
Age	18–25	30	11.9
	26–35	114	45.2
	36–44	67	26.6
	> 45	41	16.3
Marital status	Unmarried	148	58.7
	Married	104	41.3
Religion	Orthodox	150	59.5
	Muslim	87	34.5
	Other^a^	15	6.0
Ethnicity	Amhara	200	79.4
	Tigre	30	11.9
	Oromo	16	6.3
	Other^b^	6	2.4
Level of education	Diploma	18	7.1
	Degree	207	82.1
	MSc and above	27	10.7
Profession	General practitioner	37	14.7
	Medical Specialist	10	4.0
	Nurse	118	46.8
	Medical laboratory	20	7.9
	Pharmacist	31	12.3
	Midwife	20	7.9
	Other^c^	16	6.3
Service duration in year	1–5 years	122	48.4
	6–11 years	108	42.9
	> 11 years	22	8.7
Working hour per week	38–44 hr	169	67.1
	45–59 hr	47	18.7
	≥ 60 hr	36	14.3
Monthly Income(In Ethio Birr)	<=6192	43	17.1
	6193–8016	57	22.6
	8017–10,170	83	32.9
	> = 10,170	69	27.4

Key: ^a^Protestant, Catholic, ^b^Gurage, ^c^ Anesthesia, psychiatry,emergency nurse & radiography diploma

### Clinical, social support, and substance-related factors of the respondents

According to this finding, 8 (3.2%) of respondents had a history of mental illness. Among participants, 18 (7.1%) respondents had a family history of mental illness and 24 (9.5%) participants reported a history of chronic medical illness. Of these medical illnesses, hypertension 11 (4.4%), diabetes 6 (2.4%), and others were reported. From the respondents, the majority 108 (42.9%) of the healthcare professionals had received low social support. Regarding the current use of the substance, 63 (25%) of the respondents had a history of substance use within the past 3 months before data collection time. Among the users, 38 (15.1%) used alcohol, 13 (5.2%) of the respondents were chewing khat, 5 (2.0%) smoking a cigarette and 7 (2.5%) of the respondents used cannabis/pethidine within the past 3 months (Table 2).

**Table 2:** Clinical, social support, and substance-related factors of the healthcare professionals at DCSH, Dessie, Northeast, Ethiopia, 2020 (*N* = 252)

Variable	Category	Frequency	Percentage (%)
History of mental illness	Yes	8	3.2
	No	244	96.8
Family history of mental illness	Yes	18	7.1
	No	234	92.9
History of chronic medical illness	Yes	24	9.5
	No	228	90.5
Social support	Low social support	108	42.9
	Moderate social support	80	31.7
	Strong social support	64	25.4
Lifetime substance use	Yes	91	36.1
	No	161	63.9
Current substance use	Yes	63	25.0
	No	189	75.0

### Depressive symptoms status of participants

For assessing depressive symptoms, multiple responses were allowed to be made as depression can present with diverse symptoms. More than two-thirds, 176 (69.8%) of respondents did not complain of any symptoms of loss of interest. Three-fourths 200 (79.4%) of the respondents did not feel depressed, or hopeless, 178 (70.6) had no trouble falling or staying asleep, or sleeping too much, 190 (75.4%) did not feel tired and 182 (72.2%) had trouble concentrating on things, such as reading the newspaper or watching television, over the past 2 weeks (Table 3).

**Table 3:** Depressive symptoms status of the healthcare professionals at DCSH, Dessie, Northeast, Ethiopia, 2020 (*N* = 252)

Characteristics	Not at all		Several Days		More than half the days		Nearly every day	
	N	%	N	%	N	%	N	%
Little interest or pleasure in doing things	176	69.8	47	18.7	24	9.5	5	2.0
Feeling down, depressed, or hopeless	200	79.4	43	17.1	7	2.8	2	0.8
Trouble falling or staying asleep, or sleeping too much	178	70.6	40	15.9	23	9.1	11	4.4
Feeling tired or having little energy	190	75.4	31	12.3	21	8.3	10	4.0
Poor appetite or overeating	195	77.4	31	12.3	14	5.6	12	4.8
Feeling bad about yourself or that you are a failure or	217	86.1	24	9.5	8	3.2	3	1.2
Trouble concentrating on things, such as reading the newspaper or watching television	182	72.2	34	13.5	27	10.7	9	3.6
Moving or speaking so slowly that other people could have noticed. Or the opposite being so	217	86.1	24	9.5	7	2.8	4	1.6
Thoughts that you would be better off dead, or of hurting yourself	228	90.5	15	6.0	4	1.6	5	2.0

### Level of depressive symptoms of participants

**Fig. 2:**
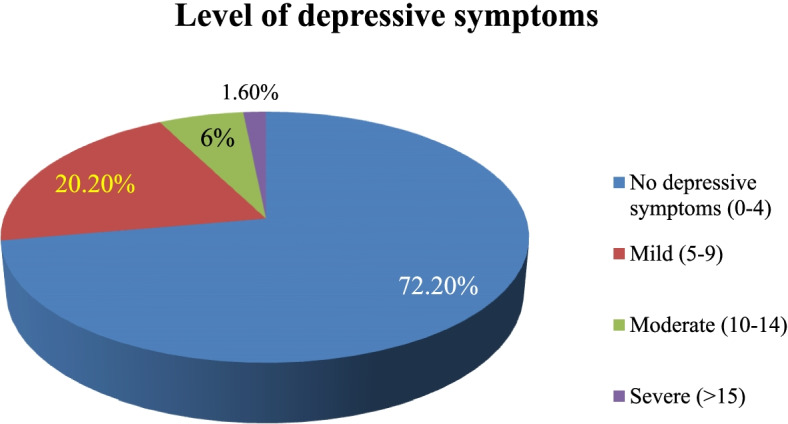
Depressive symptoms among the healthcare professionals at DCSH, Dessie, Northeast, Ethiopia, 2020 (*N* = 252)

The overall prevalence of depressive symptoms was 27.8% (95% CI:22.6,33.7). This, in the absolute figure, translates to 70 healthcare professionals with depressive symptoms. Whereas 182 (72.2%) had no reported depressive symptoms. The most frequent level of depressive symptoms was a mild (score of 5–9) depressive symptoms 51(20.2%), followed by moderate depressive symptoms (score of 10–14) 15(6%), and then severe depressive symptoms (score of > 15) 4(1.6%). This is depicted in (Fig. 2).

### Factors associated with depressive symptoms among healthcare professionals

Bivariate and multivariable logistic regression analysis was done to identify factors associated with depressive symptoms among healthcare professionals. In the bivariate analysis, sex, marital status, monthly income, service year, family history of mental illness, history of chronic medical illness, ever using the substance, current substance use, and social support showed a *p*-value of < 0.25 and became candidates for multivariable analysis. In multivariable binary logistic regression, variables; sex, marital status, family history of mental illness, and current substance use were found to be statistically associated with depressive symptoms at a *p*-value less than 0.05.

The odds of depressive symptoms were nearly twice as high among female participants as among males [AOR = 1.94; 95% CI (1.02, 3.67)]. Unmarried participants were nearly 2.16 times more likely to be married [AOR = 2.16; 95%CI (1.12,4.15)]. Likewise, those healthcare professionals who had a family history of mental illness were 7.31 times more likely to have depressive symptoms as compared with respondents who did not have a family history of mental illness [AOR = 7.31; 95%CI (2.27,23.49)]. Furthermore, the odds of having depressive symptoms among healthcare professionals who use current substances was about 2.67 times higher as compared with the referent groups [AOR = 2.67; 95%CI (1.36,5.24)] (Table 4).

**Table 4:** Bivariate and multivariable logistic regression analysis results of depressive symptoms among healthcare professionals at DCSH, Dessie, Northeast, Ethiopia, 2020 (*N* = 252)

Variables	Category	Depressive symptoms		COR(95%C.I)	AOR(95%C.I)	***P***-values
		Yes	No			
Sex	Female	30(33.7%)	59(66.3%)	1.56(0.88,2.75)	1.94(1.02,3.67)	0.042*
	Male	40(24.5%)	123(75.5%)	1	1	
Marital status	Unmarried	47(31.8%)	101(68.2%)	1.64(0.92,2.92)	2.16(1.12,4.15)	0.021*
	Married	23(22.1%)	81(77.9%)	1	1	
Monthly income	< 6192	19(44.2%)	24(55.8%)	2.62(1.15,5.96)	2.10(0.85,5.19)	0.106
	6193–8016	10(17.5%)	47(82.5%)	0.71(0.30,1.70)	0.58(0.23,1.49)	0.26
	8017–10,170	25(30.1%)	58(69.9%)	1.43(0.68,2.96)	1.51(0.68,3.35)	0.305
	> 10,171	16(23.2%)	53(76.8%)	1	1	
Service year	1–5	34(27.9%)	88(72.1%)	0.55(0.23,1.42)	0.46(0.14,1.49)	0.196
	6–10	27(25.0%)	81(75.0%)	0.48(0.18,1.25)	0.40(0.13,1.22)	0.107
	> 11	9(40.9%)	13(59.1%)	1	1	
Family history of mental illness	Yes	13(72.2%)	5(27.8%)	8.07(2.76,23.62)	7.31(2.27,23.49)	< 0.001*
	No	57(24.4%)	177(75.6%)	1	1	
Chronic medical illness	Yes	11(45.8%)	13(54.2%)	2.43(1.03,5.70)	2.049(0.78,5.29)	0.142
	No	59(25.9%)	169(74.1%)	1	1	
Ever use substance	Yes	31(34.1%)	60(65.9%)	1.61 (0.92,2.84)	0.38(0.10,1.43)	0.152
	No	39(24.2%)	122(75.8%)	1	1	
Current use substance	Yes	28(44.4%)	35(55.6%)	2.80(1.53,5.12)	2.67(1.36,5.24)	0.004*
	No	42(22.2%)	147(77.8%)	1	1	
Social support	Poor	36(33.3%)	72(66.7%)	1.63(0.80,3.30)	1.88(0.84,4.23)	0.126
	Moderate	19(23.8%)	61(76.3%)	1.02(0.47,2.21)	0.97(0.39,2.38)	0.946
	Strong	15(23.4%)	49(76.6%)	1	1	

*Statistically significant at *P*-value < 0.05, *COR* Crude Odds Ratio, *AOR* Adjusted odds Ratio, 1 = reference category, Hosmer Lemeshow goodness-of-fit 0.93, degrees of freedom = 8, Maximum VIF =2.6

## Discussion

This study aimed at estimating the prevalence of depressive symptoms among healthcare professionals in Dessie Comprehensive Specialized Hospital and identifying its correlates. The result showed that the prevalence of depressive symptoms among healthcare professionals was 27.8% (95% CI: 22.6, 33.7) with the no, mild, moderate, and severe being 72.2, 20.2, 6, and 1.6% respectively. This result was agreed upon by other studies done in Australia (32.4% [11], and Pakistani 25.8% [19]. However, the proportion of depressive symptoms, in our study was lower when compared to various studies conducted in China 38% [12], Hong Kong 35.8% [13], Saudi Arabia 43.9% [15], Bagdhad 70.25% [16], and Egypt 59% [17]. This disparity might be due to the assessment tool difference in which a previous study DASS-21 was used in Hong Kong [13], while in this study PHQ-9 was used. Also, sample size difference might be another possible reason for the incongruence in the Chinese study, in which 1320 participants were involved and all were nurses; in our study, we included participants from multiple health professions [12]. Another possible reason might be the difference in participants who had different socio-economic and demographic characteristics in the populations.

On the other hand, this study finding was higher when compared with a study done in Vietnam 13% [14], Malaysia 10.7% [18], two different studies in Nigeria 10.7 and 17.3% [20, 24]. The discrepancy might be due to the inclusion criteria. For example, in Nigeria, one study included only resident doctors using the assessment of the Mini-International Neuropsychiatric Interview (M.I.N.I) tool [20], whereas, our study included all healthcare professionals. As well, our study population is much higher than that from Malaysians, the possible reason for the discrepancy might be due to the different study settings, use of different tools, sample size, the difference in culture of study participants, and the increased workload due to the war in Ethiopia. Moreover, the possible reason for the difference may be due to study participants, time variation, working environment and probably due to the difference in a medical setting.

Regarding factors associated with depressive symptoms, in the current study, we found that being female was nearly 2 times more likely to have depressive symptoms than male sex participants. This is supported by a study conducted in Nigeria [24]. Several studies revealed that there was a sex difference in stress and coping styles, women find themselves in stressful circumstances more often than men, and their coping style is more emotion-focused than that of men [29]. Social roles also seem significant in the stressful life experiences of females, especially in low-income countries. There can be gender differences in the use of psychological coping skills in male and female healthcare workers which are important for the mitigation of depressive symptoms [30]. Furthermore, The prevalence of depressive symptoms is higher in women than in males, due to hormonal imbalance, which can be associated with childbirth, menstruation, and menopause [31].

The current study results revealed that those who were unmarried were 2.16 times more likely to have depressive symptoms as compared to those who were married. This result was in line with previous results from Saudi Arabia [23], Bagdhad [16], Egypt [17], and Nigeria [20]. Being unmarried has the psychosocial risk factors of depressive symptoms due to a lack of a partner to express their daily stressors, thereby lacking social support and social buffer [32]. In addition, it is widely believed that being married could confer an individual with better mental health and hence, mental illness morbidity [33].

Another predictor of depressive symptoms was a family history of mental illness. Those healthcare professionals who had a family history of mental illness were 7.31 times more likely to have depressive symptoms as compared with respondents who do not have a history of mental illness. This might be explained by the fact that mental illness has an inherited base, families are stigmatized and there are various types of a burden on the family members concerning financial expenses and giving care to the patient as well as the offspring might be stressed and worried about their parent’s health condition, this might be an increment for the risk of having depressive symptoms [1].

Also, in the current study, healthcare professionals who use current substances are 2.67 times more likely to experience depressive symptoms. This result was agreed to by a previous study [13]. Even if the cause and effect are not clear in this study, this result could be due to either the fact that depressed healthcare professionals are more prone to substance use to relieve themselves from the depressed mood or maladaptive substance use can alter their mood to the extent of depressive symptoms [1]. Substances use can trigger or intensify the feelings of loneliness, sadness, and hopelessness often associated with depressive symptoms among participants [34].

## Limitations

Because these findings were a cross-sectional study, it is difficult to establish a causal relationship between risk factors and depression. However, the present study provides data on depressive symptoms amongst healthcare professionals in northeast, Ethiopia. We suggest future studies on the national prevalence of depressive symptoms among hospital workers.

## Conclusion

There was a high prevalence of depressive symptoms among healthcare staff at Dessie Comprehensive Specialized Hospital. Being a female, unmarried, family history of mental illness, and current substance use were variables that were significantly associated with depressive symptoms among healthcare professionals. Therefore, they should be promptly screened and managed at a healthcare institution. In addition, a further longitudinal study including healthcare professionals from other hospitals in Dessie is warranted to have a clearer image of the situation.

## Data Availability

All data generated or analyzed during this study are included in this published article. The data sets of the current study is available from [Tamrat Anbesaw, email: tamratanbesaw@gmail.com; Mobile: + 251(0)9–11289143, Wollo University, Dessie upon reasonable request.

## Abbreviations

AOR: Adjusted Odds Ratio
CI: Confidence Interval
DALYs: Disability Adjusted Life Year
DCSH: Dessie Comprehensive Specialized Hospital
DASS-21: Depression, Anxiety and Stress scale-21
ETB: Ethiopian Birr
HCPs: Healthcare professionals
MSc: Master of Science
PHQ-9: Patient Health Questionnaires
SD: Standard Deviation
SPSS: Statistical Package for Social Science
WHO: World Health Organization
